# Point-of-care tests for influenza A and B viruses and RSV in emergency departments – indications, impact on patient management and possible gains by syndromic respiratory testing, Capital Region, Denmark, 2018

**DOI:** 10.2807/1560-7917.ES.2020.25.44.1900430

**Published:** 2020-11-05

**Authors:** Uffe Vest Schneider, Mona Katrine Alberthe Holm, Didi Bang, Randi Føns Petersen, Shila Mortensen, Ramona Trebbien, Jan Gorm Lisby

**Affiliations:** 1 Department of Clinical Microbiology, Copenhagen University Hospital Hvidovre, Hvidovre, Denmark; 2 Department of Virus & Microbiological Special Diagnostics, Statens Serum Institut, Copenhagen, Denmark

**Keywords:** Roche cobas Liat system, influenza A and B viruses and RSV, point-of-care, near patient testing, bedside testing, FilmArray, syndromic testing, patient management, indication

## Abstract

**Background:**

Point-of-care tests (POCT) for influenza A and B viruses and respiratory syncytial virus (RSV) were implemented in emergency departments of all hospitals in the Capital Region of Denmark in 2018.

**Aim:**

To establish whether POC testing for influenza viruses or RSV is based on a valid respiratory symptom indication, whether changes in patient management based on a positive result are safe and whether syndromic POC testing may benefit patients with influenza or RSV.

**Methods:**

Samples from 180 children (< 18 years) and 375 adults tested using POCT between February and July 2018 were retested for 26 respiratory pathogens. Diagnosis, indication for POC testing, hospitalisation time, antimicrobial therapy and readmission or death within one month of testing were obtained from patient records.

**Results:**

A valid indication for POC testing was established in 168 (93.3%) of children and 334 (89.1%) of adults. A positive POCT result significantly reduced antibiotic prescription and median hospitalisation time by 44.3 hours for adults and 14.2 hours for children, and significantly increased antiviral treatment in adults. Risk of readmission or death was not significantly altered by a positive result. Testing for 26 respiratory pathogens established that risk of coinfection is lower with increasing age and that POCT for adults should be restricted to the influenza and RSV season.

**Conclusion:**

Positive POCT resulted in changed patient management for both children and adults, and was deemed safe. POCT for additional pathogens may be beneficial in children below 5 years of age and outside the influenza and RSV season.

## Introduction

Over a dozen different platforms for target amplification-based point-of-care tests (POCT) are now available through several different companies [1]. Studies have evaluated the benefits of POC testing for respiratory pathogens on patient management [2-13], but their conclusions are conflicting and the benefits for patient management are therefore not fully understood. Several studies found that POC testing may improve patient management by deferring hospital admission [3], reducing hospitalisation time [4,5], improving targeted use of antiviral treatment [4,6-9,12], reducing prescription [10] and duration of antibiotic treatment [5], reducing in-hospital isolation time [8], improving use of side room isolation facilities [6] and decreasing overall costs of hospitalisation [3,5,12,13]. In contrast, other studies reported that POC testing for respiratory pathogens does not significantly reduce prescription or duration of antibiotics [4,7-9,11], do not reduce hospitalisation time or defer admissions [7-9] and do not reduce the risk of death or readmission [4,7,8].

In January 2018, routine POC testing for influenza A and B viruses and human orthopneumovirus (formerly respiratory syncytial virus, RSV) was implemented at all hospital emergency departments in the Capital Region of Denmark. Quality assurance was established by retesting the POCT samples for 20 viral and six bacterial respiratory targets at the National World Health Organization (WHO) Influenza Laboratory, Department of Virus and Microbiological Special Diagnostics, Statens Serum Institut (SSI), Copenhagen, Denmark. Accuracy and error rates of the POCT were determined for bedside use by emergency department clinical personnel or laboratory technicians (under review). Several concerns were raised [14] in connection with the implementation of POC testing for influenza A and B viruses and RSV [14]. It has been suggested that indication for POCT would increase as clinicians got access to a fast and low-complexity bedside test, which could lead to unnecessary testing and added expense without any added clinical benefit. Questions have also been raised as to whether clinicians would act on a positive test result and change patient management, and whether such changes would be safe for patients [14].

It is also unknown whether testing for additional respiratory pathogens using POCT would benefit patients and if so, which patients should be offered this testing. We aim to evaluate data for the first 6 months following the introduction of POCT in the Capital Region of Denmark to establish whether such testing is conducted based on a valid respiratory symptom indication. We also aim to examine whether clinicians safely use positive POCT results to change patient management by comparing treatment of POCT positive patients with POCT negative patients. Additionally, we identify which other viral and bacterial pathogens can be detected in the patient samples, and whether a positive syndromic test result for these pathogens could potentially influence patient management. Finally, we consider scenarios in which syndromic POCT may be of added benefit to POC testing for influenza A and B viruses and RSV in order to improve patient management.

## Methods

### Study design

This clinical impact study compared positive POCT results with negative POCT results. Consecutive patient samples (n = 555) tested for influenza A and B viruses and RSV using the cobas Liat system (Roche Diagnostics, Hvidovre, Denmark) between February and July 2018 were included in the study. The test period was restricted to the first 6 months after POCT implementation to ensure a fast assessment and allow necessary changes in instructions for clinical personnel to be implemented, and allow access to the cobas Liat instruments prior to the following influenza and RSV season from week 40 2018 to week 20 2019.

Patients were tested for influenza A and B viruses and RSV by POCT if the attending physician found the patient to be likely to have influenza A or B or RSV infection based on the clinical presentation of the patient.

### Demographics

Data regarding age at sampling, sex, sample date, time and date for admission to and discharge from hospital, initiation and type of antibacterial or antiviral treatment on admittance to hospital, readmission or death within 1 month of previous hospitalisation, lung X-ray within 24 hours of a POCT and clinical diagnosis were extracted from the patients’ electronic record. Patients were considered children if they were under 18 years of age at time of sampling.

For some patients, clinical diagnosis was established retrospectively as no diagnosis had been registered in the electronic patient record. A clinical diagnosis was established by one of the medical doctors authoring this manuscript and verified by another medical doctor. This was done by accessing the POCT result and reviewing additional clinical results documented in the electronic patient record including: lung X-ray, lung stethoscopy, temperature, oxygenation, clinical respiratory symptoms, reported pain, leucocytosis and C-reactive protein level. Other diagnostic relevant information recorded for individuals > 18 years of age was the CURB-65 score (confusion, blood urea nitrogen, respiratory rate, blood pressure and age 65 or older). For one child, no clinical diagnosis had been reported and data in their electronic patient record were limited and indicated other or no infection. Their diagnosis was therefore registered as not available.

Clinical diagnosis was recorded either as: a viral respiratory tract infection (RTI); bacterial RTI; a RTI that could not be differentiated as either bacterial or viral; another infection not originating from a respiratory focus; or no infection at all based on the clinical diagnosis in the electronic patient record.

### Indication for point-of-care testing

Indication for POC testing was considered valid if any respiratory symptoms were reported in the electronic patient record either as a patient-reported symptom or established as part of an objective examination e.g. by inspection of cavum oris or lung stethoscopy of the patient or by X-ray of the thorax.

### Point-of-care testing

Samples were collected and tested locally by emergency department clinical personnel or laboratory technicians at all four hospitals (three emergency departments and one paediatric emergency department), in the Capital Region of Denmark. After testing, the remaining sample material was sent to the Department of Clinical Microbiology at Hvidovre University Hospital and forwarded to the Danish National World Health Organization (WHO) Influenza Laboratory, Department of Virus and Microbiological Special Diagnostics, Statens Serum Institut (SSI), Copenhagen, Denmark.

### Retesting at Statens Serum Institut

At SSI, samples were tested in single real-time PCR assays targeting: *Bordetella parapertussis, Bordetella pertussis, Chlamydia pneumoniae, Chlamydia psittaci, Legionella* spp*., Mycoplasma pneumoniae,* enterovirus, human coronavirus 229E, human coronavirus HKU1, human coronavirus NL63, human coronavirus OC43, human mastadenovirus A-G (formerly adenovirus), human metapneumovirus (hMPV), human polyomavirus 3, human polyomavirus 4, human respirovirus 1 (formerly parainfluenzavirus 1), human respirovirus 3 (formerly parainfluenzavirus 3), human orthorubulavirus 2 (formerly parainfluenzavirus 2), human orthorubulavirus 4 (formerly parainfluenzavirus 4), influenza A virus either as non-typeable, subtype A(H1N1)pdm09 or A(H3N2), influenza B virus, influenza C virus, parechovirus, primate bocaparvovirus 1 + 2 (formerly bocavirus), rhinovirus and RSV. Assays are laboratory-developed tests quality assured according to the quality programme provided by the WHO. Total nucleic acids were extracted from 200 µL of patient sample after the addition of PolyA (0.05 mg/mL) as a carrier (Roche Diagnostics) by a MagNa Pure 96 extraction robot using the MagNa Pure 96 DNA Viral NA small volume kit, the ‘plasma small volume protocol’, and an elution volume of 100 µL (Roche Diagnostics). Real-time PCR was performed using either an MX3005P (Stratagene, Agilent Technologies, Glostrup, Denmark) or an ABI 7500 (Applied Biosystems, ThermoFisher Scientific, Slangerup, Denmark) real-time system. For each assay, 5 µL of extracted nucleic acids was used in a total reaction volume of 25 µL.

### Statistical analysis

Statistical analysis was performed using MS Excel, MedCalc online version (MedCalc software, Ostend, Belgium) and Social Science Statistics (socscistatistics.com). Descriptive data are reported as number and percentage of individuals. Age in years and hospitalisation time in hours are reported as median with interquartile range (IQR).

Comparison of proportions was performed by ‘N-1’ chi-squared test and comparison of medians was performed by two-tailed Mann–Whitney U test, with level of significance at p < 0.05.

### Ethical statement

Collection of patient data for quality assurance and development of treatment was granted according to Danish legislation by the board of directors at Hvidovre University Hospital (application WZ19001024–2019–30) and data were anonymised and used for statistical analysis according to the regulation.

## Results

### Basic patient characteristics and indication for point-of-care testing

Of the tested individuals, children below 18 years accounted for 32.4% (180/555), adults between 18 and 65 years accounted for 37.8% (210/555), and elderly patients above 65 years accounted for 29.7% (165/555) (Table 1).

**Table 1:** Characteristics of patients tested for influenza A and B viruses and RSV using point-of-care tests at hospital emergency departments, and indication for testing, Capital Region of Denmark, February–July 2018 (n = 555)

Characteristics	Number of children (n = 180)	%	Number of adults (n = 375)	%
**Age (years)**				
< 2	122	67.8	NA	NA
2–5	29	16.1	NA	NA
6–17	29	16.1	NA	NA
18–65	NA	NA	210	56
> 65	NA	NA	165	44
**Sex**				
Female	68	37.8	213	56.8
**Number of POCT by month**				
February	1	0.6	39	10.4
March	66	36.7	189	50.4
April	75	41.7	102	27.2
May	15	8.3	31	8.3
June	11	6.1	10	2.7
July	12	6.7	4	1.1
**Valid indication for POCT**				
February to July	168	93.3	334	89.1
February to April	135	95.1	294	89.1
May to July	33	86.8	40	88.9
**Clinical diagnosis**				
Viral RTI	124	68.9	142	37.9
Bacterial RTI	9	5.0	95	25.3
Viral or bacterial RTI	9	5.0	27	7.2
Other infection	27	15.0	58	15.5
No infection	10	5.6	53	14.1
Not available	1	0.6	0	0.0
**Antibacterial or viral treatment**				
Only oseltamir treatment	0	0.0	15	4.0
Only antibacterial treatment	33	18.3	194	51.7
Oseltamir and antibacterial treatment	0	0.0	12	3.2
**Outcome**				
Median hospitalisation time – hours (IQR)	13.5	0.0–39.7	44.7	6.8–131.7
Readmission within 30 days	43	23.9	108	28.8
Death within 30 days of discharge	0	0.0	51	13.6
Median age for death (IQR)	NA	NA	77.5	66.8–84.0

IQR: interquartile range; NA: not applicable; POCT: point-of-care test; RTI: respiratory tract infection.

Children were defined as patients under 18 years at time of presentation.

Patients were primarily tested in March and April 2018, and most patients (both children and adults) were found to have a viral or mixed viral and bacterial RTI. Median hospitalisation time for children was 13.5 hours (IQR: 0.0–39.7) and 33 (18.3%) of the children were treated with antimicrobial therapy initiated during hospital admission (Table 1). Median hospitalisation time for adults was 44.7 hours (IQR: 6.8–131.7) and 206 (54.9%) of adults were treated with antimicrobial therapy initiated during hospital admission. Twenty-seven (7.2%) of adult patients received oseltamir treatment for influenza due to a positive POCT result, and 12 of these patients were also treated with antibiotics. Approximately one quarter of all children and adults were readmitted to hospital within 30 days of the previous discharge. Fifty-one adult patients (13.6%), median age 77.5 years (IQR: 66.8–84.0), died under hospitalisation or within 30 days of discharge (Table 1). Nine of the patients who died were below 65 years of age and all had underlying conditions. Three had cancer, two had liver cirrhosis, two had Down syndrome, one had chronic obstructive pulmonary disease (COPD) and diabetes and one had severe COPD.

One hundred and sixty eight (93.3%) children and 334 (89.1%) adults were found to have a valid indication for POC testing, leaving 12 (6.7%) children and 41 (10.9%) adults tested for influenza A and B viruses and RSV using a POCT without any respiratory symptomatology recorded in their electronic patient record.

### Effects of a positive point-of-care test result on patient management

A positive POCT result for influenza A or B viruses or RSV was significantly associated with a viral or mixed viral/bacterial RTI diagnosis in both children and adults (p < 0.0001) (Table 2).

**Table 2:** Effect of a positive influenza A and B viruses and RSV point-of-care test result on patient management, Capital Region of Denmark, February–July 2018 (n = 555)

Children (n = 180)	POCT positive		POCT negative		Difference		p value
	Total n	%	Total n	%	z-score	95% CI	
Hospitalisation time, hours (IQR)	1.0	0.0–27.1	15.2	(1.4–42.2)	2.4	NA	0.017
Antibacterial treatment	2	3.8	31	24.4	20.6	9.5 to 29.2	0.0011
Readmission within one month	13	24.5	30	23.6	0.9	−11.6 to 15.5	0.897
Lung X-ray within 24 hours	2	3.8	15	11.8	8.0	−2.0 to 15.3	0.094
Viral or viral/bacterial RTI	53	100.0	80	63.0	37.0	26.6 to 45.7	< 0.0001
Bacterial RTI	0	0.0	9	7.1	7.1	−0.4 to 12.9	0.047
**Adults (n = 375)**	**POCT positive**		**POCT negative**		**Difference**		**p value**
	**Total n**	**%**	**Total n**	**%**	**z-score**	**95% CI**	
Hospitalisation time, hours (IQR)	16.3	2.6–75.3	60.6	11.3–142.2	3.9	NA	< 0.0001
Antibacterial treatment	33	28.4	161	62.2	33.7	23.0 to 43.1	< 0.0001
Oseltamir	21	18.1	6	2.3	15.8	9.3 to 23.9	< 0.0001
Readmission within one month	30	25.9	78	30.1	4.2	−5.9 to 13.4	0.403
Death within one month of hospitalisation	14	12.1	37	14.3	2.2	−5.9 to 9.0	0.563
Lung X-ray within 24 hours	70	60.3	156	60.2	0.1	−10.7 to 10.5	0.984
Viral or viral/bacterial RTI	108	93.1	61	23.6	69.6	61.3 to 75.4	< 0.0001
Bacterial RTI	6	5.2	89	34.4	29.2	21.3 to 35.8	< 0.0001

CI: confidence interval; IQR: interquartile range; NA: not applicable; POCT: point-of-care test; RSV: respiratory syncytial virus; RTI: respiratory tract infection.

Children were defined as patients under 18 years at time of presentation.

Significantly more adult patients with a positive POCT result were treated with oseltamir (p < 0.0001) and significantly fewer with antibiotics (p < 0.0001) than those adults with a negative POCT result (Table 2). An added benefit was significantly reduced hospitalisation time for adults (p < 0.0001) and children (p < 0.017) with a positive POCT result compared to a negative POCT result (a median of 16.3 hours (IQR: 2.6–75.3) vs 60.6 hours (IQR: 11.3–142.2) for adults, respectively, and a median of 1 hour (IQR: 0.0–27.1) vs 15.2 hours (IQR: 1.4–42.2) for children, respectively). Risk of readmission or death within 1 month of discharge and the likelihood of having a lung X-ray performed within 24 hours of a POCT was almost equally distributed between POCT-positive and -negative patients (Table 2).

### Additional pathogen findings by syndromic respiratory testing

When tested for the 20 viral and six bacterial pathogens included in Table 3, 56.2% (312/555) of all POCT samples were positive for one or more pathogen.

**Table 3:** Viral and bacterial targets detected, by age group, among patients who took an influenza A and B virus and RSV point-of-care test at a hospital emergency department, Capital Region of Denmark, February–July 2018 (n = 312)

Pathogen detected	Age (years)				
	0–1	2–5	6–17	18–65	> 65
*Bordetella pertussis*	1	0	0	0	0
Enterovirus	13	2	1	1	0
Human coronavirus 229E	1	0	0	0	1
Human coronavirus HKU1	0	0	0	2	0
Human coronavirus NL63	6	0	2	2	0
Human coronavirus OC43	0	0	0	1	0
Human mastadenovirus A-G	15	5	2	6	1
Human metapneumovirus	7	2	0	12	10
RSV	30	1	0	8	10
Human polyomavirus 3	1	1	0	0	0
Human polyomavirus 4	5	1	0	0	0
Human respirovirus 1	1	0	0	0	0
Human respirovirus 3	10	2	0	3	2
Influenza A virus (non-typeable)	1	0	1	4	1
Influenza A(H1N1)pdm09 virus	3	4	1	9	2
Influenza A(H3N2) virus	1	1	3	13	15
Influenza B virus	1	1	1	26	28
*Mycoplasma pneumoniae*	0	0	1	6	0
Parechovirus	1	0	0	0	0
Primate bocaparvovirus 1 + 2	3	0	0	1	0
Rhinovirus	36	2	3	12	8
Total number of detected viruses	135	22	14	100	78
Total number of detected bacteria	1	0	1	6	0
Total number of samples with detection of one pathogen	74	15	13	98	76
Total number of samples with co-detection of pathogens	28	2	1	4	1
Total number of samples tested negative for all pathogens	20	12	15	108	88
Single target detection rate %	60.7	51.7	44.8	46.7	46.1
Coinfection rate %	23.0	6.9	3.4	1.9	0.6

RSV: respiratory syncytial virus.

All samples were negative for *Bordetella parapertussis, Chlamydia pneumoniae, Chlamydia psittaci,* human orthorubulavirus 2 and 4, influenza C virus and *Legionella* spp.

In children below 2 years of age, 60.7% (74/122) were positive for one pathogen, whereas 23.0% (28/122) were positive for multiple pathogens (23 for two pathogens, four for three pathogens and one for four pathogens). The rate of coinfection of pathogens diminished with age. Among 2–5 year olds, two children were found to be positive for either three or four pathogens. From 6 years of age and older, all coinfections were caused by two pathogens (Table 3). In children below 2 years of age, the most prevalent findings were rhinovirus, RSV, human mastadenovirus A-G, enterovirus and human respirovirus 3, whereas influenza A virus and influenza B virus were only rarely detected in this age group (Table 3). Influenza B virus was predominately detected in adults and influenza A(H1N1)pdm09 virus was most prevalent in children aged 2­–5 years, with a declining prevalence in the older age groups and among the children below 2 years. Influenza A(H3N2) virus predominated in children above 5 years of age and in adults (Table 3). Seven influenza A virus-positive samples were non-typeable after amplification and sequencing at SSI. No further attempts were conducted to identify the influenza A virus subtype. The most prevalent pathogens detected between February and April were influenza B virus, influenza A virus, RSV and hMPV, all of which were not detected in May to July 2018 (Table 4).

**Table 4:** Viral and bacterial pathogens detected among patients who took an influenza A and B virus and RSV point-of-care test at a hospital emergency department, by sampling month, Capital Region of Denmark, February–July 2018 (n = 312)

Pathogen detected	Month (2018)					
	February	March	April	May	June	July
*Bordetella pertussis*	0	0	0	0	0	1
Enterovirus	0	8	3	3	1	2
Human coronavirus 229E	0	0	2	0	0	0
Human coronavirus HKU1	1	0	1	0	0	0
Human coronavirus NL63	1	6	3	0	0	0
Human coronavirus OC43	0	1	0	0	0	0
Human mastadenovirus A-G	1	12	7	5	0	4
Human metapneumovirus	0	15	16	0	0	0
RSV	1	21	27	0	0	0
Human polyomavirus 3	0	0	2	0	0	0
Human polyomavirus 4	0	2	2	1	0	1
Human respirovirus 1	0	0	1	0	0	0
Human respirovirus 3	0	3	5	7	0	2
Influenza A virus (non-typeable)	0	4	3	0	0	0
Influenza A(H1N1)pdm09 virus	1	14	3	0	0	0
Influenza A(H3N2) virus	2	23	8	0	0	0
Influenza B virus	8	41	8	0	0	0
*Mycoplasma pneumoniae*	1	3	2	1	0	0
Parechovirus	0	0	0	0	0	1
Primate bocaparvovirus 1 + 2	1	2	1	0	0	0
Rhinovirus	0	17	27	8	4	5
Total number of detected viruses	16	169	120	24	5	15
Total number of detected bacteria	1	3	2	1	1	1
Total number of samples with detection of one pathogen	17	140	88	18	5	8
Total number of samples with co-detection of pathogens	0	15	15	3	0	3
Total number of samples tested negative for all pathogens	23	100	74	25	16	5
Single target detection rate %	42.5	54.9	49.7	39.1	23.8	50.0
Coinfection rate %	0	5.9	8.5	6.5	0	18.8

RSV: respiratory syncytial virus.

All samples were negative for *Bordetella parapertussis, Chlamydia pneumoniae, Chlamydia psittaci,* human orthorubulavirus 2 and 4, influenza C virus and *Legionella* spp.

Rhinovirus, human respirovirus 3, human mastadenovirus A-G and enterovirus were detected most frequently in May to July indicating the season differences between different respiratory pathogens (Table 4).

### How syndromic respiratory testing may impact patient management

Syndromic testing for 26 other respiratory pathogens was compared with POC testing for influenza A and B viruses and RSV and is presented in Table 5. A significantly shorter hospitalisation time (1.0 vs 16.0 hours, p = 0.024) and significantly fewer antibiotics (3.8% vs 17.1%, p = 0.020) were observed for children with a positive POCT result for influenza A and B viruses and RSV compared to children positive for other respiratory viruses (Table 5). A similar effect on hospitalisation time in adults was not observed, even though those with a positive POCT result for influenza A and B viruses and RSV received significantly fewer antibiotics (p < 0.0001) compared with adults positive for other viruses (Table 5).

**Table 5:** Potential effect of point-of-care testing for influenza A and B viruses and RSV, and syndromic testing for 26 other respiratory viruses on patient management, Capital Region of Denmark, February–July 2018 (n = 312)

Children (n = 133)	Influenza A and B and RSV POCT positive		Positive for other respiratory viruses^a^		Difference		p value
	Total n	%	Total n	%	z-score	95% CI	
Hospitalisation time, hours (IQR)	0.7	(0.0–37.0)	15.7	(1.5–36.1)	2.52	NA	0.012
Antibacterial treatment	2	4.1	14	16.7	12.6	1.0 to 22.4	0.032
Readmission within one month	12	24.5	18	21.4	3.1	−10.9 to 18.6	0.685
Lung X-ray within 24 hours	2	4.1	11	13.1	9.0	−2.1 to 18.3	0.093
Viral or viral / bacterial RTI	49	100.0	65	77.4	22.6	12.1 to 32.6	0.0003
Bacterial RTI	0	0.0	4	4.8	4.8	−3.1 to 11.6	0.122
**Adults (n = 179)**	**Influenza A and B and RSV POCT positive**		**Positive for other respiratory viruses^a^**		**Difference**		**p value**
	**Total n**	**%**	**Total n**	**%**	**z-score**	**95% CI**	
Hospitalisation time, hours (IQR)	17.5	(3.6–76.7)	41.6	(3.4–105.5)	-2.03	NA	0.042
Antibacterial treatment	35	31.5	40	58.8	27.3	12.3 to 40.8	0.0003
Antiviral treatment	20	18.0	0	0.0	18.0	10.0 to 26.2	0.0002
Readmission within one month	27	24.3	19	27.9	3.6	−9.1 to 17.2	0.592
Death within one month of hospitalisation	14	12.6	8	11.8	0.8	−10.1 to 10.2	0.867
Lung X-ray within 24 hours	64	57.7	43	63.2	5.6	−9.2 to 19.6	0.461
Viral or viral / bacterial RTI	100	90.1	29	42.6	47.4	33.7 to 59.3	< 0.0001
Bacterial RTI	8	7.2	24	35.3	28.1	16.0 to 40.5	< 0.0001

IQR: interquartile range; NA: not applicable; POCT: point-of-care test; RSV: respiratory syncytial virus; RTI: respiratory tract infection.

^a^ Patients positive for other respiratory viruses that are typically included in syndromic POCT platforms excluding influenza A virus, influenza B virus and RSV.

Children were defined as patients under 18 years at time of presentation.

## Discussion

The risk of excessive use of POCT for detecting respiratory pathogens is frequently mentioned as a concern when considering placing POCT for bedside use by clinical personnel in emergency departments. To our knowledge, no studies have previously looked at the indications for POC testing when implemented for routine clinical patient management. In our study, 93.3% of tested children and 89.1% of tested adults had clinical signs of a respiratory infection and thereby a valid indication for POC testing. Interestingly, 33 children (18.3%) and 40 adults (10.7%) were tested in May to July 2018 even though no influenza A virus-, influenza B virus- or RSV-positive patients were detected after week 20 2018. It is therefore recommended to restrict the use of POCT for respiratory testing to relevant time periods based on the seasonal variation of the pathogens included in the assay used for the POCT [15]. It may also be beneficial to introduce an electronic prompt question for clinical signs of respiratory infection when ordering the POCT to increase the likelihood of a valid indication for POCT.

Previous studies agree that POCT for respiratory infections improve the targeted use of antiviral treatment [4,6,8,9,12], which is also supported by the present study. In contrast, the effect on prescription and duration of antibiotic treatment and hospitalisation time is debatable [4,5,7-11]. Most studies support that prescription and duration of antibiotic treatment is not significantly reduced by POC testing [4,7-9], and three studies found no reduction in duration of hospitalisation [7-9]. These studies looked at the effect of a POCT intervention using the Biofire FilmArray PCR system (Biomerieux, Saint-Louis, US), which analyses samples in central laboratories [4,7-9]. This method is in contrast to the present study, where POC testing was conducted bedside using the much faster cobas Liat influenza A and B viruses and RSV assay. In addition, the present study compared the effect of POCT on antibiotic prescription and duration of hospitalisation.

The present study showed that clinicians do take the POCT result into account when diagnosing the patient and deciding treatment strategy. Even though hospitalisation time was shortened and antibiotic use was decreased, we found no difference in readmission or mortality between POCT-positive and -negative patients, indicating that the use of a POCT for clinical decisions regarding hospital admission and initiation of antibiotic therapy is safe for the patient. Other studies have reported that a general introduction of syndromic POCT for multiple respiratory pathogens without taking the result of the POCT into account does not alter prescription and duration of antibiotic treatment or duration of hospitalisation [4,7-9,11]. One may speculate that a fast and positive result for other respiratory pathogens will also impact prescription of antibiotics and duration of hospitalisation, as is suggested by our data, as these lesser pathogenic viruses are often treated with antibiotics and patients are admitted even though they are diagnosed with viral or mixed viral and bacterial RTI.

Coinfections with several respiratory pathogens have frequently been found in younger children and have been shown to reduce with increasing age [15,16], which is in line with the present study. The use of syndromic POCT should therefore be considered in children below 5 years of age and may also be beneficial outside the appropriate season for targeted POCT for influenza A and B viruses and RSV. Further randomised controlled trials are needed to clarify whether positive syndromic POCT for respiratory viruses can significantly reduce antibiotic prescription and duration of hospitalisation compared to targeted testing for influenza A and B viruses and RSV. Such studies are relevant as 322 patients, or 58.0% of all patients tested by POCT in the present study, were positive for one or more of the 26 tested respiratory pathogens.

The present study has several limitations including that patients were only tested if the treating physician suspected the patient to be positive for influenza viruses or RSV based on clinical evaluation. The prevalence of respiratory viruses is therefore expected to be higher in our sample than in the general population. Samples were collected consecutively, but only from the middle of the RSV and influenza season, which may have influenced patient handling and the detection of other viral and bacterial pathogens. This study compares POCT-positive and -negative samples and cannot be used to evaluate whether patient management was changed by the introduction of POCT compared to centralised laboratory testing. In addition, we can only hypothesise whether syndromic POC testing may result in changed patient management compared with POC testing for influenza A and B viruses and RSV.

It is still unknown how the introduction of POCT will influence influenza and RSV surveillance in Denmark. As results are reported directly into the national microbiology database, it may impact surveillance data if testing frequency and indication for testing changes over time. As the present POC testing for influenza A and B viruses and RSV does not subtype influenza A virus-positive isolates, it may influence national surveillance as most centralised microbiology laboratories report influenza A virus subtypes. Most POCT samples will not be subtyped as only a fraction of samples will be subtyped by SSI in the future. Further studies are therefore needed to establish how POCT for influenza A and B viruses and RSV are changing our national surveillance data.
